# Exercise training improves long-term memory in obese mice

**DOI:** 10.1093/lifemeta/load043

**Published:** 2023-11-15

**Authors:** Oliver K Fuller, Casey L Egan, Tina L Robinson, Nimna Perera, Heidy K Latchman, Lauren V Terry, Emma D McLennan, Carolina Chavez, Emma L Burrows, John W Scott, Robyn M Murphy, Henriette van Praag, Martin Whitham, Mark A Febbraio

**Affiliations:** Monash Institute of Pharmaceutical Sciences, Monash University, Melbourne, Victoria, 3052, Australia; Monash Institute of Pharmaceutical Sciences, Monash University, Melbourne, Victoria, 3052, Australia; Monash Institute of Pharmaceutical Sciences, Monash University, Melbourne, Victoria, 3052, Australia; Monash Institute of Pharmaceutical Sciences, Monash University, Melbourne, Victoria, 3052, Australia; La Trobe Institute for Molecular Science, La Trobe University, Melbourne, Victoria, 3086, Australia; Monash Institute of Pharmaceutical Sciences, Monash University, Melbourne, Victoria, 3052, Australia; Monash Institute of Pharmaceutical Sciences, Monash University, Melbourne, Victoria, 3052, Australia; Florey Institute of Neuroscience and Mental Health, University of Melbourne, Melbourne, Victoria, 3052, Australia; Florey Institute of Neuroscience and Mental Health, University of Melbourne, Melbourne, Victoria, 3052, Australia; Monash Institute of Pharmaceutical Sciences, Monash University, Melbourne, Victoria, 3052, Australia; La Trobe Institute for Molecular Science, La Trobe University, Melbourne, Victoria, 3086, Australia; Department of Biomedical Sciences, Charles E. Schmidt College of Medicine, and Stiles-Nicholson Brain Institute, Florida Atlantic University, Jupiter, FL 33458, United States; College of Life and Environmental Sciences, University of Birmingham, Edgbaston, Birmingham B15 2TT, United Kingdom; Monash Institute of Pharmaceutical Sciences, Monash University, Melbourne, Victoria, 3052, Australia

**Keywords:** obesity, exercise, cognition

## Abstract

Obesity has been linked to a range of pathologies, including dementia. In contrast, regular physical activity is associated with the prevention or reduced progression of neurodegeneration. Specifically, physical activity can improve memory and spatial cognition, reduce age-related cognitive decline, and preserve brain volume, but the mechanisms are not fully understood. Accordingly, we investigated whether any detrimental effects of high-fat diet (HFD)-induced obesity on cognition, motor behavior, adult hippocampal neurogenesis, and brain-derived neurotrophic factor (BDNF) could be mitigated by voluntary exercise training in male C57Bl/6 mice. HFD-induced impairment of motor function was not reversed by exercise. Importantly, voluntary wheel running improved long-term memory and increased hippocampal neurogenesis, suggesting that regular physical activity may prevent cognitive decline in obesity.

## Introduction

Obesity, a significant risk factor for cardiometabolic disease, may play a role in neurodegenerative diseases such as dementia [1–4]. Evidence from animal models suggests that obesity leads to increased cognitive dysfunction, particularly in hippocampal-dependent processes, including learning and spatial memory [5, 6]. In humans, many studies have shown that obesity is linked to cognitive dysfunction, even when cognitive aging is controlled [7, 8]. It has been suggested that this relationship is due to the sensitivity of the hippocampus to changes in diet, since consuming a Western-style diet (typically characterized by high intakes of saturated fats, refined sugars, and a reduced intake of fiber, fruits, and vegetables) leads to impairments in memory retention [9], reduced hippocampal volume [10, 11], impairments in motor coordination [12, 13], and increased cerebral white-matter atrophy [14]. The mechanisms underlying the link between the consumption of high-fat diet (HFD) and impairment in hippocampus-dependent processes are not well understood, but several factors have been proposed, including impaired plasticity through a reduction in brain-derived neurotrophic factor (BDNF) levels [15], chronic increases in inflammatory cytokines [16, 17], increased apoptosis [18], and neuronal demyelination [19] in the central nervous system (CNS).

Higher fitness levels are associated with maintenance or improvements to brain biology and function by targeting a range of pathways, including those potentially involved in HFD-induced cognitive impairment. Associations between cortical gray matter volume, cardiovascular fitness, and memory function in young human adults have been observed [20]. In healthy older adults, cardiovascular fitness was associated with faster and more accurate spatial short-term memory performance. In this study, the walking group showed a 1%–2% increase, whereas the controls showed a 1%–2% decrease in hippocampal volume over the 1-year intervention [21]. Exercise remains one of the most effective treatments for dementia, particularly Alzheimer’s disease (AD), the risk of which is reduced by 45% [22]. A mechanism through which exercise can elicit improvements in memory function is adult hippocampal neurogenesis [23].

Assessing neurogenesis in human subjects is complex and controversial [24–26]. Consequently, the phenomenon of hippocampal neurogenesis is primarily studied in rodents. Running has been shown to enhance neurogenesis within the dentate gyrus (DG), more than doubling the production of new neurons in young [27] and aged mice [28]. While the mechanism(s) underlying these positive effects is (are) poorly understood, neurotrophins such as BDNF are important, since mice lacking BDNF show decreased synaptic plasticity in the hippocampus, cortex, and striatum [15, 29], and lack of running-induced neurogenesis in the absence of the tyrosine receptor kinase B (TrkB) in adult neural stem cells (NSCs) [30]. BDNF gene transcription and protein expression increase within the rodent hippocampus after exercise [31–34]. In obese human subjects, high-intensity exercise leads to higher levels of circulating BDNF compared with normal-weight individuals [35] Whether this translates to an increase in BDNF in the brain or improved cognitive performance is unclear [36]. Since consumption of an HFD is associated with reduced levels of BDNF [37], this may be a mechanism through which exercise can improve cognitive dysfunction observed in obesity.

Exercise can also modulate inflammation via the production of pro- and anti-inflammatory cytokines. The biological effect of such production is, however, complex. High levels of tumor necrosis factor (TNF) production promote apoptotic cell death and impair proliferation and remyelination [38, 39]. In animal models, consumption of an HFD and obesity are associated with elevated levels of pro-inflammatory cytokines in the brain. These are directly linked to deficits in hippocampus-dependent memory [40, 41], suggesting chronic inflammation plays a role in mediating performance in hippocampus-dependent tasks, primarily through synaptic dysfunction [17]. On the other hand, relatively small increases in pro-inflammatory cytokines, including TNF, reportedly benefit adult hippocampal neurogenesis and nerve remyelination [39, 42]. Whether voluntary running mediates this effect through the acute low-level release of pro-inflammatory factors involved in promoting adult hippocampal neurogenesis and reducing apoptosis, or through the regulation of myelination remains to be fully elucidated.

While neuronal apoptosis within the hippocampus and cortex is commonly associated with AD [43], mounting evidence suggests that even short-term HFD feeding can increase apoptosis within the hippocampus of obese animal models [18, 19]. While the effect of HFD-induced apoptosis within the hippocampus on cognitive performance is not well established, the effect in the hypothalamic arcuate nucleus (ARC), responsible for energy homeostasis, is well characterized, with both short- and long-term HFD feeding inducing neuronal apoptosis in mice [44–46]. This contributes to the development of obesity through the loss of proopiomelanocortin neurons, which regulate food intake, leading to increased weight gain and further neuronal death [47, 48]. Both forced and voluntary running have been shown to reduce neuronal apoptosis within the hippocampus and hypothalamus [49–51]. Reducing hippocampus apoptosis may provide a pathway through which exercise can benefit hippocampus-dependent processes in cases of HFD-induced cognitive impairment.

Accordingly, in the present study, we examined (i) whether HFD-induced obesity caused cognitive impairment; (ii) whether voluntary exercise training (VET), in the form of voluntary wheel running, could ameliorate any impairments; (iii) the mechanism(s) for the observed phenotypes in mice. We show that voluntary running improves long-term memory and adult hippocampal neurogenesis in middle-aged male, obese C57BL/6 mice. Moreover, these effects were likely mediated through an exercise-induced increase in hippocampal transcription of *Tnf*, BDNF expression, and phosphorylation of the classical mitogen-activated protein (MAP) kinase (MAPK) extracellular signal-regulated kinases (ERK) in the hippocampus, which regulates neurogenesis within this brain region.

## Results

### Diet and VET influence energy homeostasis in mice

**Figure 1:**
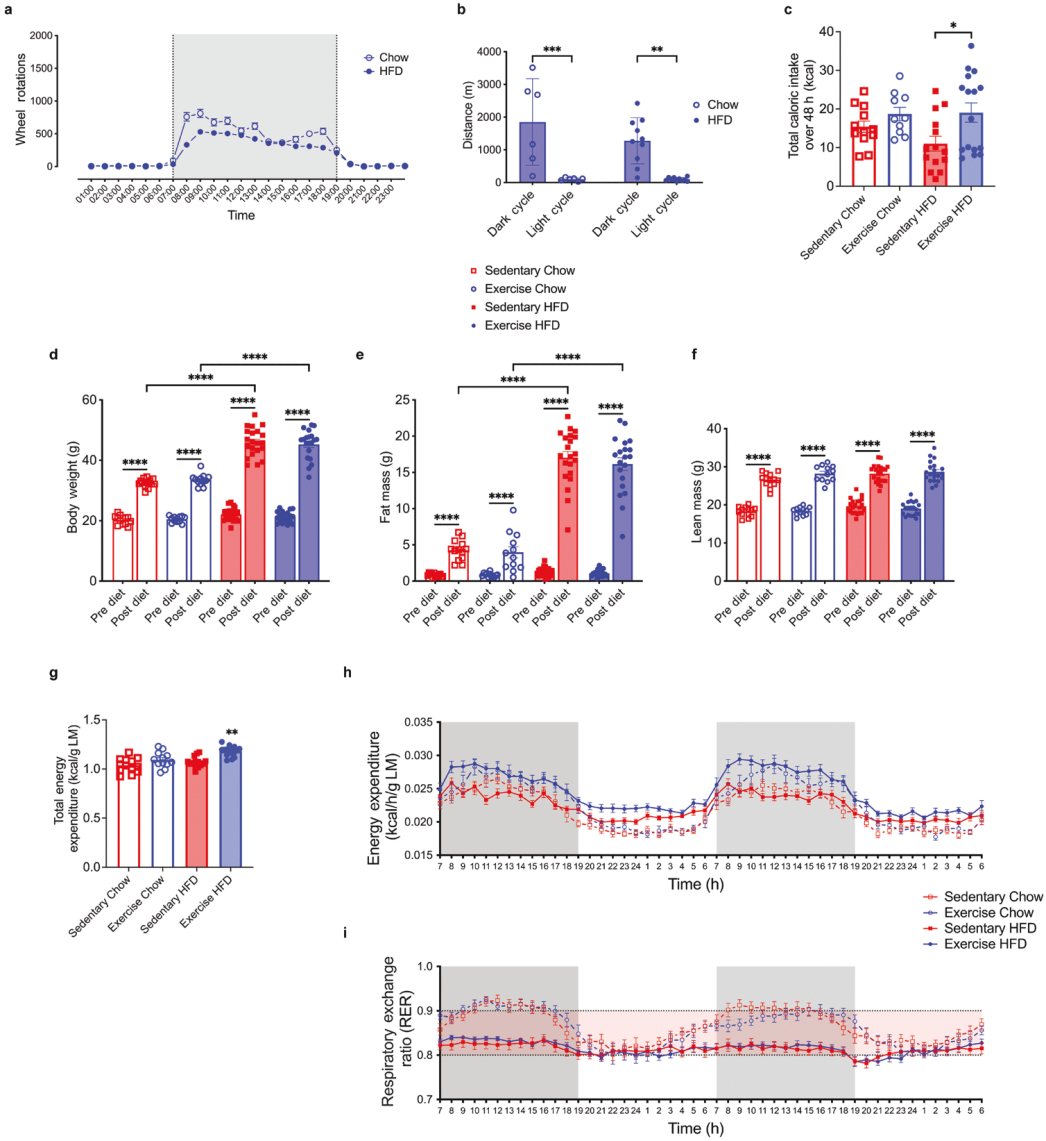
Diet and VET influence energy homeostasis in mice. (a) Running wheel data shown as wheel rotations for a 24-h cycle averaged over 3 weeks (11–13 weeks old). (b) Total distance over 24 h, averaged over 3 weeks for both dark and light cycles. Significance was calculated using mixed-effects analysis, *P* < 0.01, ^***^P < 0.001. HFD, *n* = 20; Chow, *n* = 12. (c) Total caloric intake over the same 48-h period. (d) Body weight, (e) Absolute fat mass, (f) Absolute lean mass pre-diet (Week 0) and post-diet (Week 20). Statistical significance was calculated using two-way ANOVA Tukey *post hoc*. ^*^P < 0.05, ^****^P < 0.0001. (g) Total energy expenditure over 48 h normalized by lean mass. Statistical significance was calculated using ANCOVA (main effect of exercise *F*_1,51_* *= 24.88, *P* < 0.0001. Diet *F*_1,51_* *= 3.079, *P* = 0.0853. RER *F*_1,51_* *= 2.304, *P* = 0.1352). Pairwise comparison: Exercise HFD is significantly different from all other conditions (^**^P < 0.01). (h) Averaged energy expenditure (EE) (binned hourly) over the 48-h period normalized to lean mass. Statistical significance was calculated using RM ANOVA Tukey *post hoc*. (i) Averaged RER (binned hourly) over the 48-h period using RM ANOVA Tukey *post hoc*. Exercise HFD, *n* = 20; sedentary HFD, *n* = 21; exercise chow, *n* = 12; sedentary chow, *n* = 13. All data are displayed as group mean ± SEM.

Male C57BL/6J mice were fed a chow diet (12% of total energy from fat) or an HFD (43% of total energy from fat) at 6 weeks of age. After 6 weeks of dietary intervention, mice were dual housed with two running wheels that were either locked (Sedentary; Sed) or unlocked (VET) and studied for a further 14 weeks (Supplementary Fig. S1a). On average, mice in the VET groups ran 1500 m/day (HFD) and 2000 m/day (chow diet), respectively (Fig. 1a and b). As expected, mice in the VET groups ran more (*P* < 0.01) in the dark compared with the light cycle, while mice ran less in HFD VET compared with Chow VET, although this was not statistically significant (Fig. 1a and b). After 12 weeks of experimental intervention, mice were placed in metabolic chambers (Promethion® metabolic phenotyping system) for 5 days. After a 3-day acclimatization period (due to the necessity to singly house the mice), caloric intake and energy expenditure data were collected over the final 2 days. Caloric intake did not differ when comparing Chow VET with Chow Sed. In contrast, caloric intake was greater (*P* < 0.05) when comparing HFD VET with HFD Sed (Fig. 1c). Body weight and body composition were monitored weekly during the experimental intervention using quantitative magnetic resonance (echoMRI™) (Supplementary Fig. S1b–d). Body weight, fat mass, and lean mass were increased (*P* < 0.05) in all groups, indicative of growth and maturation of all mice during the 20-week intervention (Fig. 1d–f, Supplementary Fig. S1b–d). Consumption of an HFD increased (*P* < 0.0001) body weight (Fig. 1d) and total fat mass (Fig. 1e) to a greater magnitude in both VET and Sed groups compared with chow-fed groups. Somewhat surprisingly, VET did not reduce body mass or fat mass irrespective of diet (Fig. 1d and e). In addition, VET did not increase lean mass to a greater extent than the animals that had locked wheels, irrespective of diet (Fig. 1f). There was a small, but statistically significant increase in total energy expenditure when comparing HFD VET with the other three groups (*P* < 0.01), which were almost identical (Fig. 1g). Energy expenditure and respiratory exchange ratio (RER) binned hourly over the 48-h measurement period are shown in Fig. 1h and i, respectively. Taken together, these data indicate that consumption of an HFD markedly increases body mass and fat mass, but running ~1500 m/day is not an effective weight loss strategy in male C57BL/6 mice, because it results in a marked (~2-fold) increase in total caloric consumption.

### VET improves long-term memory in obese mice

**Figure 2:**
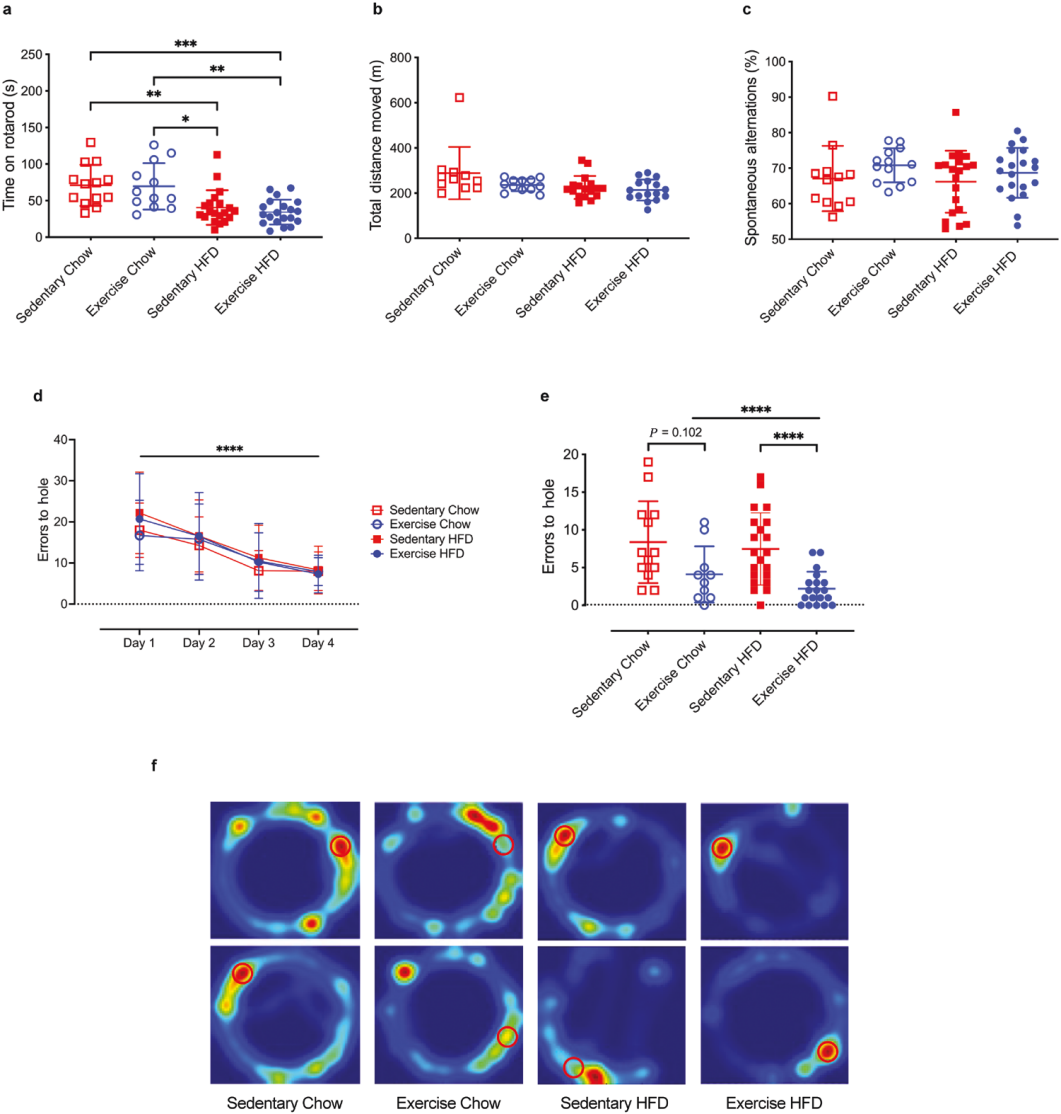
VET does not ameliorate diet-induced motor impairment or affect short-term memory, but does improve long-term memory in obese mice. (a) Rotarod results for all four experimental groups. (b) Total distance moved during LMA test. (c) Percentage of spontaneous alternations during a spontaneous alternation Y maze test. (d) Errors to hole during Barnes maze learning phase. (e) Errors to target hole achieved during probe test conducted using a Barnes maze. (f) Representative heatmaps from two mice per group during probe test. The location of the target hole is indicated by red circles. Statistical significance was calculated using two-way ANOVA Tukey *post hoc* (a–c) and negative binomial regression Tukey *post hoc* (d and e). ^*^P < 0.05, ^**^P < 0.01, ^***^P < 0.001, ^****^P < 0.0001. Exercise HFD, *n* = 20; sedentary HFD, *n* = 21; exercise chow; *n* = 12; sedentary chow, *n* = 13. All data are displayed as group mean ± SEM.

To evaluate the effects of consumption of an HFD and/or VET on motor and cognitive function, mice underwent a suite of behavioral tests in the final 2 weeks of the intervention (Supplementary Fig. S1a). In the initial test, mice were placed on a rotarod to evaluate balance, grip strength, and motor coordination. Time on the rotarod was decreased (*P* < 0.001) by HFD relative to chow diet, irrespective of VET (Fig. 2a). Hence, consumption of an HFD impaired balance, grip strength, and motor coordination, but VET was unable to rescue this, suggesting that impaired motor coordination was driven by HFD-induced increases in body weight and fat mass combined with decreases in lean mass, which were unaffected by VET (Fig. 1d–f). We next performed a locomotor activity (LMA) test to evaluate ambulatory and spontaneous LMA changes. Mice were placed into a white box for 90 min and the total distance traveled was measured. As shown in Fig. 2b, LMA was unaffected by either diet or VET. We next placed mice in the spontaneous alternation Y maze to assess short-term, working memory. Mice typically prefer to investigate a new arm of the maze rather than returning to one previously visited. This maze tests several memory systems associated with various brain regions (including the hippocampus and the prefrontal cortex). The percentage of spontaneous alternations (the number of sequential arm entries divided by the total number of arm entries) was not different between the groups (Fig. 2c), indicating that neither diet nor VET affects short-term, working memory. Of note, somewhat consistent with data obtained for the rotarod, the total distance covered in the Y maze over 90 min was decreased (*P* < 0.05) by consumption of HFD irrespective of VET (Supplementary Fig. S1e), but this did not affect spontaneous alternations. Finally, mice underwent Barnes maze test, a hippocampus-dependent task where animals learn the relationship between distal cues in the surrounding environment and a fixed escape location [52]. The experimental setup and visual cues are shown in Supplementary Fig. S1f. To validate the test, we first performed a scopolamine intervention test. Scopolamine is a muscarinic cholinergic receptor antagonist, known to impair learning and memory in mice [53]. Untreated and scopolamine-treated (1 mg/kg delivered via intraperitoneal (i.p.) injection, once daily) 12-week-old male C57BL/6J mice (*n* = 5, per group) undertook 4 days of learning with 4 days of trials. As expected, error to target hole for the probe test (number of incorrect nose pokes until the target hole is reached), conducted 3 days after the last day of learning, demonstrated that scopolamine treatment increased (*P* < 0.001) error to hole compared with the untreated group, which effectively learned the location of the target hole over the 4 days of learning phase (Supplementary Fig. S1g and h). Once validated, we subjected the experimental mice to the Barnes maze. Mice fed on an HFD moved less than those on a chow diet irrespective of VET (Supplementary Fig. S1i), but more importantly, all four groups of mice were equally effective in learning the task during the initial 4 days of learning phase (Fig. 2d). Three days after the fourth day of learning, the probe test was conducted. No differences were observed when comparing Sed Chow with Sed HFD (Fig. 2e and f). Importantly, however, VET decreased errors to hole in HFD-fed mice (*P* < 0.0001), indicating that VET improves long-term memory in obese mice. Taken together, our data indicate that consumption of an HFD that increases body weight and fat mass can impair motor coordination. More importantly, however, VET can markedly improve long-term memory in mice fed an HFD.

### VET increases BDNF and decreases TNF gene expression in the hippocampus

**Figure 3:**
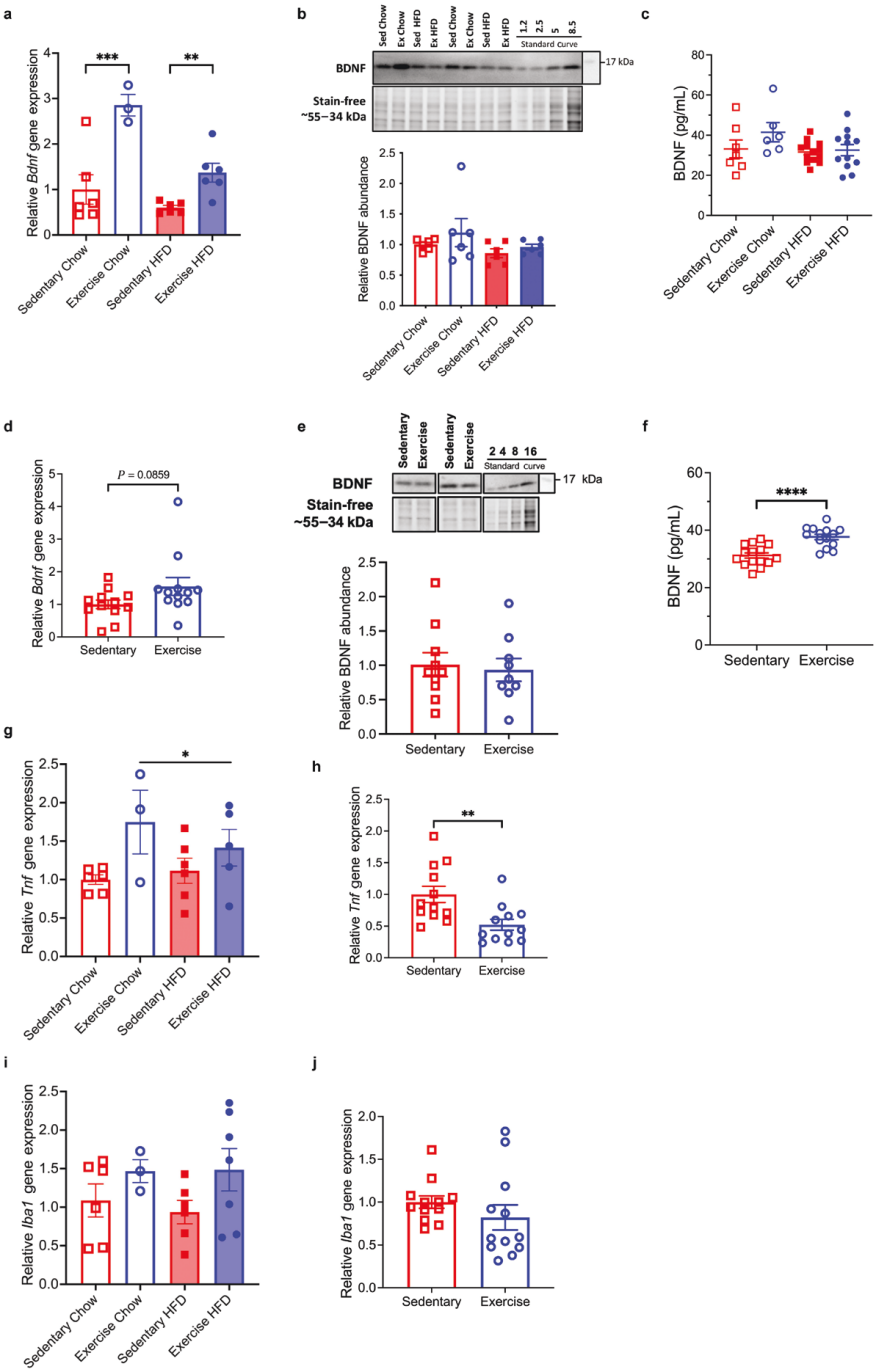
VET increases expression of *Bdnf* and decreases *Tnf* gene expression in the hippocampus but does alter BDNF protein levels in the hippocampus or circulation significantly. (a) Relative *Bdnf* gene expression in the hippocampus was measured by real-time qRT-PCR, normalized to *ActB* mRNA expression for each of the four groups, and normalized to sedentary chow group to obtain relative fold expression. (b) Western blot analysis of hippocampal BDNF. Quantification below, normalized to sedentary chow group. (c) Plasma levels of BDNF in mice who underwent VET measured at the study end point (26 weeks old). (d) Relative *Bdnf* gene expression in the hippocampus of 8-week-old male C57BL/6 mice after 60 min of forced exercise (treadmill) and sedentary controls was measured by real-time qRT-PCR, normalized to *ActB* mRNA expression, normalized to sedentary group to obtain relative fold expression. (e) Western blot analysis of BDNF expression in the hippocampus of 8-week-old male C57BL/6 mice after 60 min of forced exercise (treadmill) and sedentary controls. Quantification below, normalized to the sedentary group. (f) Plasma levels of BDNF in 8-week-old male C57BL/6 mice after 60 min of forced exercise (treadmill) and sedentary controls. (g–j) Relative *Tnf* gene expression in the hippocampus of mice after VET (g) and acute treadmill training (h), and relative *Iba1* gene expression after VET (i) and acute treadmill training (j), normalized to *ActB* mRNA expression for all groups, normalized to the sedentary group to obtain relative fold expression. Data are presented as individual and mean ± SEM (*n* = 3–16), with representative western blots and corresponding total protein stain-free images. Black lines separate noncontiguous lanes from the same gel. Statistical significance was calculated using unpaired *t*-test (two groups) and two-way ANOVA with Tukey *post hoc*. ^*^P < 0.05, ^**^P < 0.01, ^***^P < 0.001, ^****^P < 0.0001.

To elucidate the mechanisms underlying the VET-induced improvement in long-term memory in obesity, we investigated a range of biological processes within the hippocampus associated with long-term memory. Irrespective of diet, VET markedly increased the expression of *Bdnf* mRNA (*P* < 0.01) in the hippocampus (Fig. 3a). Despite this observation, we were unable to detect an increase in BDNF protein expression in the hippocampus (Fig. 3b). While there was a tendency for an increase in circulating BDNF levels with VET (*P* = 0.07) compared with Sed in chow diet-fed mice (Fig. 3c), the results were not statistically significant. Increases in BDNF expression in the hippocampus have been shown to be transient and increase within a limited time window [54]. In our experimental setup, mice underwent voluntary wheel running, so it was not possible to assess the precise time from the cessation of VET until the collection of the hippocampus at the termination of the experiment. Accordingly, to assess whether acute exercise could affect BDNF and other signaling protein levels, we performed a separate experiment where mice underwent 60 min of acute treadmill running. Upon cessation of the exercise, mice were immediately sacrificed, and the hippocampus and blood were collected and frozen. *Bdnf* mRNA levels in the hippocampus did not significantly change between sedentary and acutely exercised mice (Fig. 3d). Consistent with our results with VET, BDNF protein expression in the hippocampus was unchanged by acute exercise (Fig. 3e). In contrast, in this experiment, acute exercise markedly increased (*P* < 0.001) BDNF protein expression in the circulation (Fig. 3f). We also measured the tyrosine protein kinase B (TrkB), which is the receptor for BDNF in the hippocampus. TrkB expression was unaffected by diet, VET, or acute exercise (Supplementary Fig. S2a–f). Taken together, our results show that VET increases *Bdnf* mRNA levels in the hippocampus of obese mice, but does not significantly impact BDNF protein levels in the hippocampus or blood. In contrast, acute exercise does not change *Bdnf* mRNA expression or BDNF protein within the hippocampus, but it does increase levels of circulating BDNF.

We also measured the mRNA expression of *Tnf* and ionized calcium-binding adapter molecule 1 (*Iba1*), a calcium-binding protein that plays an important role in the functional change of microglia [55]. Irrespective of diet, VET increased *Tnf* mRNA expression (Fig. 3g). In contrast, acute exercise decreased *Tnf* mRNA expression (*P* < 0.01) (Fig. 3h). No effect of either VET (Fig. 3i) or acute exercise (Fig. 3j) was observed in relation to the *Iba1* mRNA expression. These data indicate that VET may be a mild inflammatory stimulus in the brain that may contribute to the improved memory phenotype observed in the mice.

### Acute exercise increases ERK phosphorylation in the hippocampus

**Figure 4:**
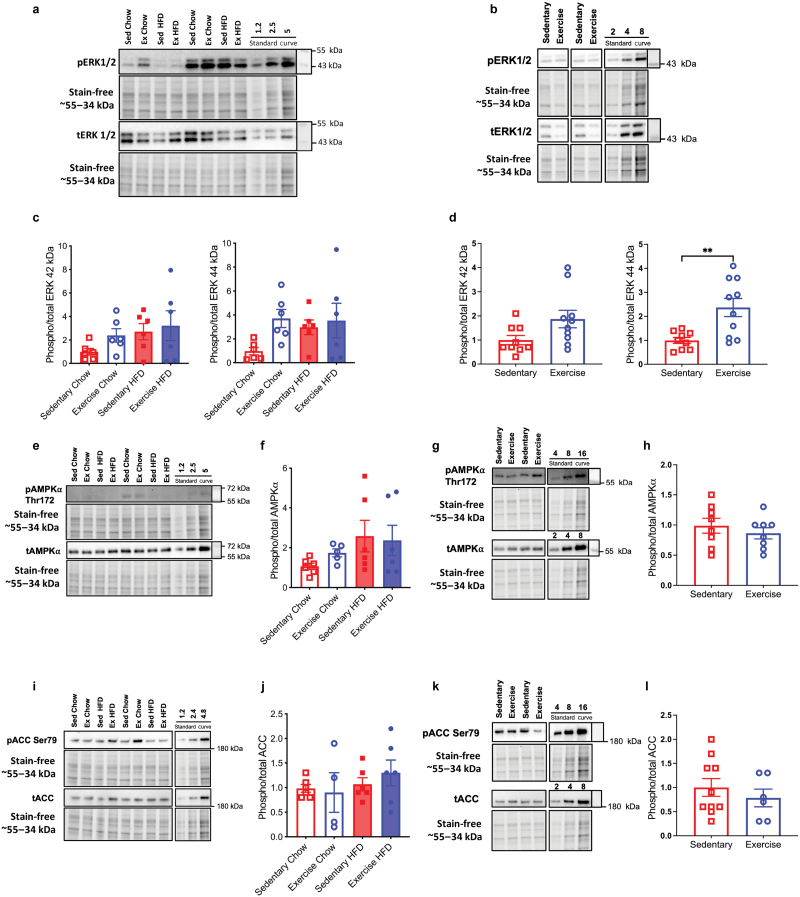
VET does not affect phosphorylation levels of ERK, AMPK, or ACC whereas acute exercise increases ERK phosphorylation in the hippocampus of mice. (a and c) Western blot analysis of phospho/total ERK 1/2 in the hippocampus of mice after VET (a) and quantification normalized to sedentary chow group (c). (b) Western blot analysis of phospho/total ERK 1/2 in the hippocampus of acute treadmill exercised mice and sedentary controls. (d) Protein abundance quantification, normalized to sedentary group for blot shown in (b). (e and f) Phospho/total AMPK alpha isoform (AMPK⍺) in the hippocampus of mice after VET (e), and protein abundance quantified and normalized to the sedentary group (f). (g and h) Western blot analysis of phospho/total AMPK⍺ in the hippocampus of acutely (treadmill) exercised and sedentary control mice (g), quantified and normalized to sedentary group (h). (i and j) Phospho/total ACC in the hippocampus of mice after VET (i), and protein abundance quantified and normalized to the sedentary group (j). (k and l) Western blot analysis of phospho/total ACC in the hippocampus of acutely (treadmill) exercised and sedentary control mice (k), quantified and normalized to sedentary group (l). Data were statistically appraised by unpaired *t*-test (two groups) and two-way ANOVA with Tukey *post hoc*. ^**^P < 0.01. Data are presented as individual and mean ± SEM (*n* = 4–9), with representative western blots and corresponding total protein stain-free images. Black lines separate noncontiguous lanes from the same gel.

VET did not affect the 42 kDa isoform of ERK irrespective of diet, or the 44 kDa ERK isoform in chow-fed conditions (*P* = 0.1599) (Fig. 4a and c). In contrast, acute exercise may increase phosphorylation of the 42 kDa ERK isoform (*P* = 0.0550), while it markedly increased the phosphorylation of the 44 kDa isoform of ERK in the hippocampus of the mice (*P* < 0.01) (Fig. 4b and d). These data suggest that exercise can increase hippocampal ERK phosphorylation, a necessary component of long-term memory in mice. Neither VET nor diet affected phosphorylation of AMP-activated protein kinase (AMPK) nor its downstream target acetyl-CoA carboxylase (ACC) in the hippocampus (Fig. 4e–l).

### VET increases neurogenesis in the hippocampus

**Figure 5:**
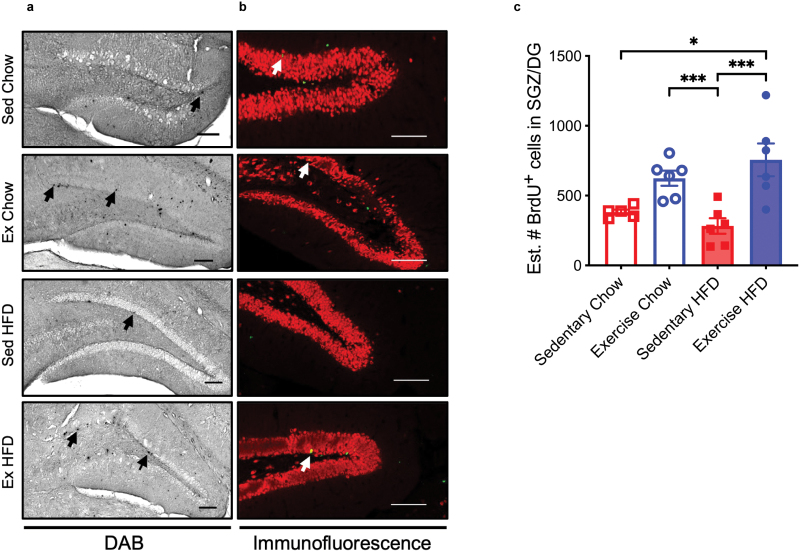
VET increases neurogenesis in the DG of mice irrespective of diet. (a) DAB staining showing BrdU-positive cells 4 weeks after last BrdU injection. Black arrows indicate BrdU-positive cells. (b) Confocal images of immunofluorescence double-stained sections. BrdU (green), NeuN (red). White arrows indicate BrdU-labeled neurons. Scale bar = 100 µm. (c) Estimated (optical fractionation) number of total BrdU-positive cells per DG, quantified from DAB stained sections. Statistical significance was calculated using two-way ANOVA Tukey *post hoc*. ^*^P < 0.05, ^***^P < 0.001. Exercise HFD, *n* = 6; sedentary HFD, *n* = 6; exercise chow, *n* = 6; sedentary chow, *n* = 6. All data were displayed as group mean ± SEM.

We next investigated whether consumption of an HFD and/or VET affected cell survival and differentiation in the DG, since neurogenesis is strongly implicated in long-term memory. 3,3ʹ-diaminobenzidine (DAB) staining was undertaken on brain sections 4 weeks after the last injection of bromodeoxyuridine (BrdU). Representative images for each group are shown in Fig. 5a, with examples of BrdU-positive cells within the DG indicated with a black arrow. The number of BrdU-positive cells within the DG for each animal was quantified using the optical fractionator technique and presented in Fig. 5c. Irrespective of diet, VET markedly increased the number of BrdU-positive cells in the DG (*P* < 0.0001). To confirm the differentiation of the BrdU-positive cells, sections were dual-stained for BrdU and the neuronal marker neuronal nuclei (NeuN). Representative confocal images with BrdU, labeled neurons indicated by a white arrow are shown in Fig. 5b. To assess the effect of HFD feeding and/or VET exercise on hippocampus apoptosis, the terminal deoxynucleotidyl transferase (TdT) dUTP nick end labeling (TUNEL) staining was undertaken on sections containing DG (Supplementary Fig. S2g). Neither diet nor VET affected apoptosis in this region of the brain (Supplementary Fig. S2h).

### Proteomic analysis of VET in HFD-fed mice

To gain insight into other factors that may be important in mediating cognitive improvement with VET, we performed proteomic analysis on the hippocampi of HFD-fed mice. We identified 5532 unique proteins present in three or more samples, with an average of 5079 proteins identified per sample. Protein abundance spanned a range of ~4.52 orders of magnitude, with the least abundant protein registering an intensity value of 2.19 × 10^7^ (calcium voltage-gated channel subunit alpha1 H, CACNA1H, which is known to be lowly expressed in neuronal cells in the hippocampus) and the most abundant reaching 7.24 × 10¹¹ (β-actin, ACTB). The principal component analysis revealed an overlap of data points from the sedentary and exercise groups, indicating a lack of distinct separation and suggesting that the variance within the dataset did not prominently discriminate between the two groups (Supplementary Fig. S3a). With a false discovery rate (FDR) of 0.05, no proteins differed significantly between the exercise and sedentary hippocampal samples (Supplementary Fig. S3b). Using less conservative filtering criteria (FDR set to 1), 411 proteins were identified that differed between the groups. The top 10 proteins significantly increased in the exercise vs. sedentary samples are shown in Supplementary Fig. S3c. The top seven significantly enriched pathways are shown in Supplementary Fig. S3d. Unsurprisingly, pathways associated with metabolism were the most highly enriched when comparing exercise with sedentary in HFD-fed animals.

## Discussion

Herein, we investigated whether consumption of an HFD impaired hippocampus-dependent processes, including spatial navigation, memory, and adult hippocampal neurogenesis, and whether voluntary exercise could ameliorate these deficits. We demonstrate that exercise improves long-term memory using the Barnes maze, a well established and sensitive test for assessing hippocampus-dependent processes such as spatial memory. To understand the mechanism, we assessed the expression of genes and proteins known to be involved in adult neurogenesis, neuronal survival, and remyelination. We observed an exercise-induced increase in hippocampal transcription of *Tnf* and *Bdnf* expression, and phosphorylation of ERK, the classical MAPK, in the hippocampus, all of which contribute to increased neurogenesis within this region of the brain.

In the present study, exercise increased adult hippocampal neurogenesis in HFD mice. In sedentary HFD mice, however, adult neurogenesis was not reduced. This finding is consistent with previous research in male C57Bl/6 mice [56].

Exercise promotes NSC activation and proliferation [57], spurs morphological maturation [58], and enhances survival, differentiation, and integration of new neurons [59, 60]. In mouse models of AD, both voluntary and forced exercise have been observed to foster neurogenesis within the hippocampus [61–63]. However, it is worth noting that in some studies, the increase was less pronounced than in wild-type mice [64, 65].

BDNF is abundant in the CNS and mediates key neuronal functions such as differentiation, growth, synapse formation, and neuroplasticity [66]. Several studies have reported an increase in BDNF protein expression in the hippocampus of rodents following exercise [67, 68]. In addition, although skeletal muscle releases BDNF after exercise, it has not been confirmed whether it can be released into circulation [69]. Given these previous results and the robust increase we observed in hippocampal *Bdnf* mRNA expression with exercise (Fig. 3a), we were surprised that neither VET nor acute exercise increased hippocampal BDNF protein expression (Fig. 3b and e). However, in a recent elegant study where BDNF expression was assessed in the hippocampus by bioluminescence imaging, the authors observed that the BDNF signal following a single bout of exercise is very transient and also somewhat difficult to measure [54]. As we only assessed BDNF at two time points, it is possible that we missed this transient increase. Additionally, since the mice were housed in pairs, it is challenging to distinguish the running activity of individual mice; thus, some may not have run as greatly, leading to variability in the BDNF response.

Previous studies have observed that activation of the MAPK cascade is required both in the induction of a long-lasting form of hippocampal synaptic plasticity [70] and learning in rodents [71, 72]. While exercise-induced increases in ERK phosphorylation in skeletal muscle are well established [73, 74], whether the same occurs in the hippocampus has not been widely studied. Two previous studies have demonstrated that exercise increases phosphorylation of ERK in the rat hippocampus [75]. Our data in mice support these previous reports in rats and suggest that ERK phosphorylation may be a mechanism for the VET-induced increase in neurogenesis and long-term memory. Indeed, some works on exercised mice have previously suggested the intricate interaction between signaling molecules. Specifically, previous studies have reported an increase of BDNF and phosphorylation of protein kinase B (p-Akt) in the mouse cortex [76] and demonstrated activation of the ERK-Akt-cyclic adenosine monophosphate responsive element binding protein (CREB)-BDNF pathway, indicating the potential for a robust model integrating these molecular actors [77]. However, it is pertinent to note that in our study, we did not observe increases in CREB phosphorylation (p-CREB) (data not shown), consistent with our findings showing no change in BDNF protein levels, necessitating a degree of caution when extrapolating these findings into a consolidated mechanistic framework.

As discussed, small increases in TNF within the hippocampus can influence adult hippocampal neurogenesis and nerve remyelination [39, 42]. Irrespective of diet, VET increased *Tnf* mRNA expression (Fig. 3g). Whether this contributed to VET-induced increase in neurogenesis and long-term memory is speculative, since we did not measure protein expression, while *Tnf* mRNA expression in response to an acute exercise bout actually decreased (Fig. 3h). As discussed, short-term HFD feeding has been shown to increase apoptosis within the hippocampus of obese animal models [18, 19]. Conversely, exercise reduces neuronal apoptosis within the hippocampus and hypothalamus [49]. In our study, however, we found neither diet nor VET affected apoptosis within the DG.

The cellular energy sensor AMPK has also been shown to play a critical role in the age-related decline in hippocampal neurogenesis [78], while swimming exercise increases AMPK signaling to suppress apoptosis and inflammation in aging hippocampus [79].

One limitation of our study is that we did not measure the effect of either consumption of an HFD or VET on cerebral blood flow (CBF). Thinking and learning increase CBF while reduced CBF in response to cognitive tasks is associated with poorer outcomes [80]. Furthermore, obesity decreases regional CBF [81]. Accordingly, exercise training-induced improvements in CBF have been previously observed [82] and hence we cannot rule this out as a possible mechanism for our observed phenotype. Several other factors remained unexplored in our study, including the effect of diet quality differences between HFD and chow diets. These dietary distinctions might independently impact physiological and cognitive outcomes [83]. Furthermore, we did not delve into the significance of muscle mitochondrial efficiency. Not investigating the role of muscle mitochondrial efficiency, particularly considering that mitochondria are highly sensitive to diet and exercise interventions, introduces potential confounding factors [84]. Lastly, the influence of eating schedules, recognized to affect metabolic health and systemic physiology, was not accounted for in this work.

In summary, we demonstrate that VET improves long-term spatial memory, due to increased neurogenesis in the DG. The increase in neurogenesis was possibly mediated by increased expression of *Bdnf* and *Tnf* mRNA as well as increased phosphorylation of ERK in the hippocampus. We, therefore, demonstrate that exercise training not only provides benefits to cognitive performance in healthy subjects but also protects against diet-induced impairment and neurodegeneration, which is increasingly becoming an area of focus for neurogenerative conditions such as AD.

## Materials and methods

### Subjects, housing conditions, diet, and body composition

Sixty-five 6-week-old male C57BL/6 mice were fed an HFD (43% energy from lipids, Specialty Feeds SF04) or regular chow (12% energy from lipids). Experiments were conducted using male mice to maintain data consistency and avoid potential variations associated with female reproductive cycles. At 12 weeks old, mice were divided into four groups (Sedentary chow, *n *= 14. Exercise chow, *n* = 10. Sedentary HFD, *n* = 21. Exercise HFD, *n* = 20), dual housed with two running wheels (locked for sedentary, unlocked for exercise), and studied for further 16 weeks. Wheel distance was monitored continuously. Body composition was assessed weekly using magnetic resonance imaging (echoMRI™, TX, USA).

### Acute exercise

Eight-week-old C57BL/6 mice were divided into two groups: exercise and sedentary. In the exercise group, the mice were familiarized with a treadmill over a period of 3 days (Day 1, 10 m/min; Day 2, 12 m/min; Day 3, 14 m/min). After 3 days, they participated in a 60-min session until exhaustion, where the speed increased from 10 to 24 m/min by 2 m/min every 10 min. Exhaustion was determined as the point when the mice were unable to maintain the selected running speed, even with gentle encouragement using a brush for 5–10 s. Upon reaching exhaustion, the mice were exposed to 4% isoflurane until they became unconscious. After cervical dislocation, the brain was removed.

### Metabolic phenotyping

At 20 weeks old, mice were metabolically phenotyped using the Promethion® metabolic cage system (Sable Systems International, NV, USA). Mice were acclimatized to single housing for 3 days before being placed in the system and measured for 2 days.

### Rotarod

At 24 weeks old, mice were acclimatized to the rotarod test 1 day before the measurements. This was done by having each mouse undergo two 2-min sessions on the rotarod set at a speed of 4 rpm (revolutions per minute) followed by a 2-min session with speed slowly increasing to a maximum of 40 rpm. On the following day, the trial was carried out, which involved each mouse undergoing three 5-min sessions with the rotarod speed slowly increasing to 40 rpm. The time spent on the rotarod for each run was measured and then averaged over the three runs to give the time spent on the rotarod for each mouse.

### LMA test

At 25 weeks old, mice were placed in the behavioral testing room to habituate for 1 h, two mice per cage with free access to food and water. They were then placed in a white box (38 cm × 38 cm × 38 cm), and their activity was recorded using an overhead video camera for 90 min. Their movement was tracked live using Viewer 3 (Biobserve, Germany).

### Spontaneous alternation Y maze

Mice were removed from their home cage and placed into one of the arms of the Y maze (the starting arm chosen randomly for each mouse). Cues of black and white shapes were placed in each of the arms. Fresh wood shavings were placed on the floor of the Y maze. An overhead video camera tracked the movement. White overhead lights were set to dim. Tracking software (Viewer 3, Biobserve, Germany) was activated immediately after the mouse was placed into the maze. The spontaneous alternation behavior was recorded for 10 min. An arm entry was defined when the head and shoulders of the mouse cross the threshold of the central zone and into the arm, and the animal’s snout was oriented toward the end of the arm. A spontaneous alternation was defined as a sequential entry into the three arms. After each mouse, wood shavings were discarded, and the maze was thoroughly cleaned with 70% ethanol and F10 disinfectant.

### Barnes maze

A validation study was undertaken to ensure that the Barnes maze protocol was working correctly. Untreated and scopolamine-treated (1 mg/kg delivered via intraperitoneal injection, once daily) 12-week-old male C57BL/6 mice (*n* = 5, per group) undertook 4 days of learning with four daily trials. Error to target hole for the probe test, the amount of incorrect nose pokes until the target hole was reached, conducted 3 days after day 4 of the learning phase, showed significant impairment in the scopolamine-treated group compared with the untreated group (*P* < 0.001), which effectively learned the location of the target hole over the 4-day learning phase (Supplementary Fig. S1a). This is demonstrated clearly in representative heatmaps, with the untreated control group spending the majority of the 90-s probe trial around the target hole (indicated by a red circle) compared with the scopolamine-treated animal (Supplementary Fig. S1b).

Mice were habituated on day 1 by placing them in the maze for 5 min and in the safe house for 1 min (location opposite from that used in the acquisition trial). The safe house was attached for the acquisition trials at a different location from that used for the habitation trial (different location for each mouse, not directly in line with any of the cues to reduce saliency). The position of the safe house then remained at this fixed location relative to spatial cues in the room (four 2D colored shapes on each of the walls and two 3D cues in the corners of the room, all equal distance from the Barnes maze (see Supplementary Fig. S1c) for the duration of the training period. The training consisted of 4 acquisition trials/day (3 min limit per trial, intertrial interval 15 min). Bright lights above the maze acted as the adverse stimuli. These were switched on after the mouse was released from the bucket. The trial concluded once the mouse entered the safe house, or 3 min elapsed. Three days after the final session of acquisition training, mice underwent a 90-s probe trial in which the safe house was removed from the apparatus. The probe trial was administered similarly to the acquisition trials, with a time limit of 90 s. Mice undertook the Barnes maze at 27 weeks of age. The errors to the target hole were calculated for each mouse over the learning phase and probe trial. Heatmaps were generated using ezTrack [85].

### Quantitative reverse transcriptase PCR

Total RNA was extracted from ~10 mg of hippocampal sample using TRIzol (ThermoFisher Scientific, MA, USA) according to the manufacturer’s protocol. Reverse transcription was performed using the High-Capacity cDNA reverse transcription kit (Applied Biosystems, MA, USA). Taqman fluorogenic primer probes were used to measure transcripts. The following primers were used, *Bdnf* (Mm00432069_m1)*, Iba1* (Mm00479862_g1), and *Tnf* (Mm00443258_m1) normalized to *ActB* (Mm02619580_g1) using the ΔΔC_t_ method [86].

### Western blot

Total protein from the right hippocampus for the chronic and both hippocampi for the acute blots was separated on 4%–15% Criterion Stain Free gels (Bio-Rad, CA, USA) along with 4–5 amounts of a mixed hippocampal homogenate, which was used as a calibration curve. After transferring to nitrocellulose and a series of washes, including antibody probes, protein bands were visualized using West Femto chemiluminescent substrate (ThermoFisher Scientific, MA, USA). Images were collected, and densitometry was performed using the Chemidoc MP system and Image Lab version 5.2 (Bio-Rad, CA, USA). Total protein on the gels was imaged before transfer (Stain Free imager, Bio-Rad), and western blot signals of given proteins were normalized to total protein, with both total protein and protein of interest expressed relative to their respective calibration curves.

### Enzyme-linked immunosorbent assay

The levels of plasma BDNF were quantified by enzyme-linked immunosorbent assay (ELISA) (Human/Mouse BDNF DuoSet ELISA, R&D Systems, MN, USA). All experiments were performed according to the manufacturer’s specifications.

### BrdU injections

Twenty-three-week-old mice were injected intraperitoneally daily with 50 mg/kg BrdU (Sigma-Aldrich, MO, USA), dissolved in 0.9% NaCl and filtered sterile at 0.2 μm at a concentration of 10 mg/mL for 10 days. Four weeks after the last injection, mice were humanely euthanized via I.P. injection of 65 mg/kg sodium pentobarbital (Virbac, NSW, Aus). Mice were perfused transcardially with 0.9% NaCl. The brains were removed, and the hemispheres were separated for biochemical and immunohistochemical analysis.

### Immunohistochemistry

Hemispheres intended for immunohistochemistry were post-fixed for 2 weeks in 4% paraformaldehyde at 4°C, after which they were transferred to 30% sucrose before cryosectioning. All coronal slices (40 µm) were stored at −20°C in cryoprotectant (ethylene glycol and glycerol in 0.1 mol/L phosphate buffer). Every sequential sixth tissue section between Bregma −1.28 and −3.80 mm (encompassing the DG) was selected for free-floating immunohistochemistry, along with DNA denaturation, which was carried out as described previously, with donkey serum replacing horse serum [87].

### Neurogenesis

Chromogenic detection of BrdU was identified using the VECTASTAIN® Elite ABC-HRP and DAB Substrate kits (PK-6100 and SK-4100, both Vector Laboratories, Newark, CA, USA) after initial probing with rat anti-BrdU antibody (ab6326, 1:500; Abcam, Cambridge, UK) and Biotin-SP-conjugated AffiniPure Donkey Anti-Rat IgG (#712-065-153, 1:500; Jackson ImmunoResearch, PA, USA). Slices were placed onto gelatin-coated slides, dehydrated, and mounted before imaging. Double immunofluorescent labeling for BrdU and NeuN was implemented using the BrdU antibody as before and recombinant Anti-NeuN [EPR12763] (ab177487, 1:500; Abcam, Cambridge, UK) and incubated for 72 h with their respective secondary antibodies Alexa Fluor® 488 AffiniPure Donkey Anti-Rat IgG (H + L) and Cy™3 AffiniPure Donkey Anti-Rabbit IgG (H + L) (#712-545-153 and #711-165-152, both 1:500; Jackson ImmunoResearch, PA, USA), respectively. Slices were then mounted onto gelatin-coated slides.

### TUNEL assay

Apoptosis in the brain was determined using the HRP-DAB TUNEL Assay Kit (ab206386, Abcam, Cambridge, UK) as per the manufacturer’s instructions.

### Stereology

BrdU-positive cells in the DG were counted in a one-in-six series. On a Zeiss Axio Imager M2 widefield microscope (Carl Zeiss AG, Germany), the subgranular zone (SGZ) area was traced using a 5× objective, and BrdU-positive cells were counted with a PL Neofluar40×/1.3 oil immersion objective using the optical fractionator method on Stereo Investigator® (MBF Bioscience, VT, USA). Random sampling was done using counting frames measuring 100 µm × 100 µm × 30 µm (X × Y × Z) and a guard zone of 5 µm. A grid size of 1000 µm × 1000 µm was used. Cells in the uppermost focal plane and intersecting the exclusion boundaries of the counting frame were not counted. DG sectional volume was determined by multiplying the traced section by the distance between sections to estimate the total number of BrdU-positive cells per sample. Fluorescent sections were imaged with a Leica SP8 Lightning confocal microscope (Lecia Microsystems, Hesse, Germany) at 20×/0.75 objective and analyzed in ImageJ (National Institutes of Health, MD, USA) to determine the colocalization of BrdU and NeuN labeled cells in the DG. TUNEL-stained sections and liver-positive control were imaged with an Olympus BX60 widefield microscope (Evident Corporation, Japan).

### Proteomic analysis of hippocampal tissue

One hippocampus from each animal was lysed in denaturing lysis buffer containing 6 mol/L urea, 2 mol/L thiourea, and 0.1% SDS in 0.1 mol/L HEPES. Lysates were precipitated in ice-cold methanol and chloroform and resuspended in lysis buffer. Lysates were sonicated 3 × 30 s tip probe, vortexed for 10 min, and spun at 20,000 *g* for 20 min before quantification using a Qubit fluorometer (Thermo Fisher Scientific, USA). Samples were normalized to total protein and volume in lysis buffer before the reduction in 10 mmol/L DTT for 1 h, shaking at 800 rpm. Samples were then alkylated in 25 mmol/L indole-3-acetic acid (IAA), protected from light, and shaken at 800 rpm for 1 h. Samples were quenched in the same volume of DTT followed by digestion in LysC (Wako) at a 1:50 ratio for 5 h at room temperature. Samples were diluted in 5 volumes of 0.1 mol/L HEPES and further digested in trypsin (modified, Promega) overnight at 37°C at a ratio of 1:50 with 1 mmol/L CaCl_2_ added to aid digestion. Samples were acidified to a final concentration of 1% trifluoroacetic acid and desalted on in-house made SDB-RPS (3M Empore) stage tips. Peptides were resuspended in loading buffer containing 2% acetonitrile, 0.5% acetic acid, and loaded onto a 50 cm × 75 mm inner diameter column packed in-house with 1.9 mm C18AQ particles (Dr Maisch GmbH HPLC) using an Easy nLC-1000 UHPLC operated in single column mode loading at 700 bar. Peptides were separated using a 180 min linear gradient at a flow rate of 200 nL/min using buffer A (0.1% formic acid) and a 5%–30% buffer B (80% acetonitrile, 0.1% formic acid). BSA samples were run before and during the acquisition with minimum intensity thresholds monitored throughout. Mass spectrometry (MS) data were acquired on a Q Exactive HF-X (Thermo Fisher Scientific, USA) operated in data-dependent mode. MS spectra were acquired at 70,000 resolution, m/z range of 300–1750, and a target value of 3e6 ions, maximum injection time of 100 ms. The top 20 precursor ions were isolated for MS/MS spectra after fragmentation with 2 m/z isolation, 8.3e5 intensity threshold, normalized collision energy of 30 at 17,500 resolution at 200 m/z, a 60 ms injection time, and a 5e5 AGC target. Searching of raw mass spec files and statistical analyses were carried out using Maxquant version 2.0.1.0 and Perseus v 1.6.5.0. FDR of 0.05 and 1 were used. Pathway analysis was carried out using GeneCodis 4 [88], on the significantly differed proteins (*P* < 0.05) when the FDR was set to 1 (note, potentials that 100% are false positives).

### Statistical analysis

Ordinary one-way, two-way, and repeated measures (RM) ANOVA and ANCOVA were performed in GraphPad Prism version 9.2.0 for Mac (GraphPad Software, CA, USA). Specific comparisons were made with Tukey’s *post hoc* test. Barnes maze probe trial data were non-normally distributed and thus were analyzed using a negative binomial regression model using R software (R Foundation for Statistical Computing, Austria). Pairwise comparisons were made with Tukey’s *post hoc* test. Outliers, as determined using the ESD (extreme studentized deviation) method (with a significance level set to 0.05), were excluded from the analysis.

## Supplementary Material

load043_suppl_Supplementary_Figures_S1-S3

load043_suppl_Supplementary_Figure_S1

load043_suppl_Supplementary_Figure_S2

load043_suppl_Supplementary_Figure_S3

## Data Availability

All study data are included in the article and/or supplementary information. Materials are available upon request.
