# Supplementary material for: Exercise training improves long-term memory in obese mice
Source: Life Metab. 2023 Nov 15;3(1):load043. doi: 10.1093/lifemeta/load043 (PMC11749366; doi:10.1093/lifemeta/load043)

**Supplementary Figure S1** (a−d) Experimental timeline (a); Body weight (b); Fat mass (c); andLean mass (d) as a percentage of total body weight. Access to unlocked/locked wheels was given at 6 weeks, indicated by the arrow. Statistical significance was calculated using mixed-effects analysis. ^****^*P* < 0.0001. (e) Distance moved during spontaneous alternation Y maze. (f) Top, screen grab from video stream of mouse completing Barnes maze. Bottom, Barnes maze cues, 2D shapes on walls, and 3D cues placed in the corner of the room. (g) Errors made before reaching target hole probe trial undertaken to validate the Barnes maze. Treated with scopolamine, *n* = 5; untreated, *n* = 5. Statistical significance was calculated using negative binomial regression as data non-normally distributed. ^***^*P* < 0.001. (h) Representative heatmaps for control and scopolamine-treated mice during probe trial. The target hole is shown in red. (i) Distance traveled during probe trial for each group. Statistical significance was calculated using two-way ANOVA Tukey post hoc. Exercise HFD, *n* = 20; sedentary HFD, *n* = 21; exercise chow, *n* = 10; sedentary chow, *n* = 14. All data is displayed as group mean ± SEM.


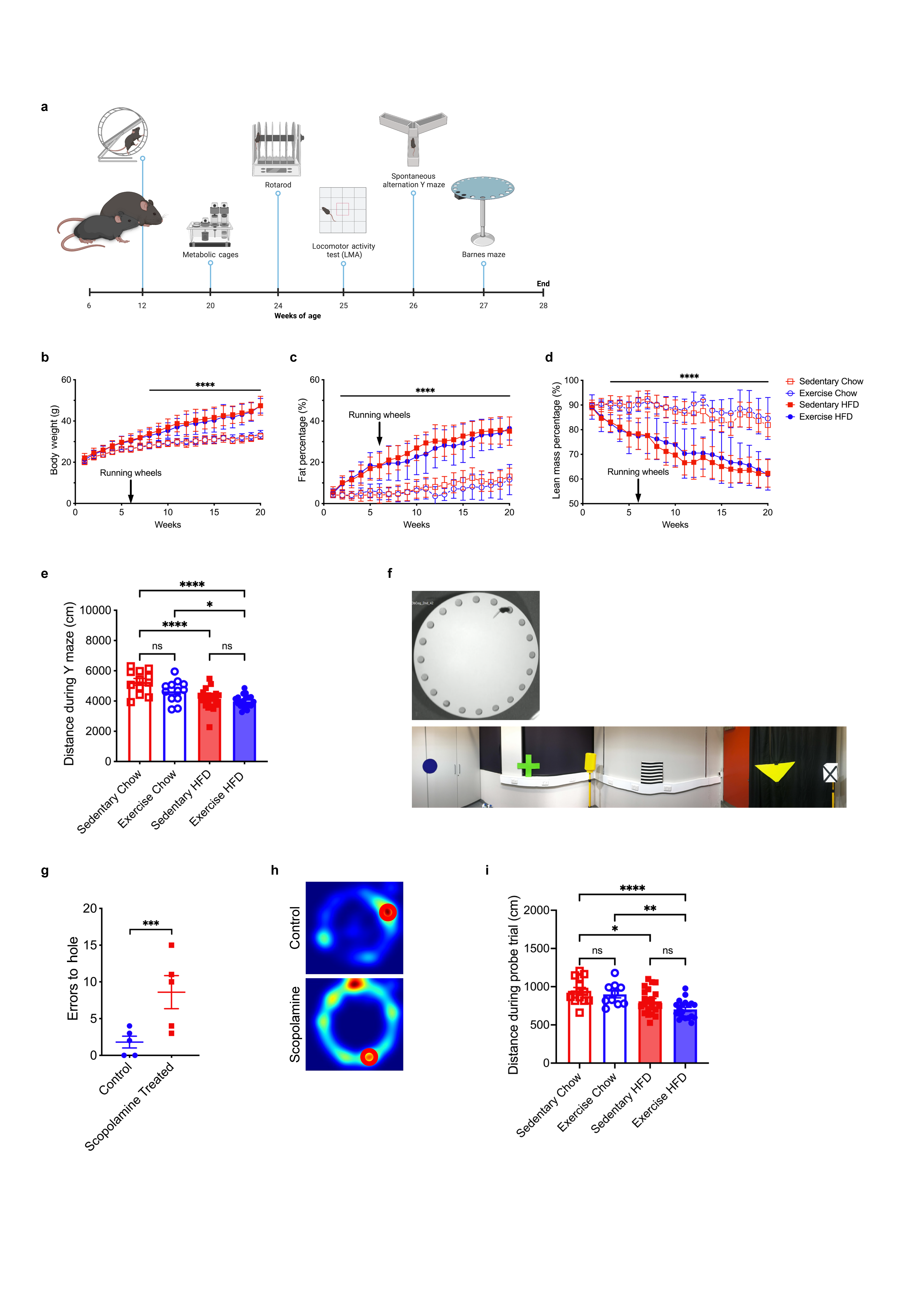


**Supplementary Figure S2** Levels of proteins associated with neurogenesis and metabolic function in hippocampal tissue of 26-week-old male C57/BL6 mice fed chow diet and HFD after 14 weeks of voluntary running and 8-week-old male C57/BL6 mice after 60 min of forced exercise (treadmill) and sedentary controls. Whole hippocampal homogenates were analyzed by western blot. (a) Representative blot for TrkB levels in the hippocampus of mice fed HFD/chow with or without running wheels. Note the same gel as BDNF blot in Figure 3b. (b and c) quantification of TrkB blot shown in (a). (d) Hippocampal TrkB levels in 8-week-old male C57/BL6 mice after 60 min of forced exercise. (e and f) quantification of TrkB blot shown in (d). (g) Representative images of TUNEL stained DG for each group and liver positive control. Black arrows indicate TUNEL-positive cells. Scale bar = 50 µm. (h) Quantification of TUNEL-positive cells in the DG, *n* = 3 for each group. Statistical significance was calculated using two-way ANOVA. All data is displayed as group mean ± SEM.


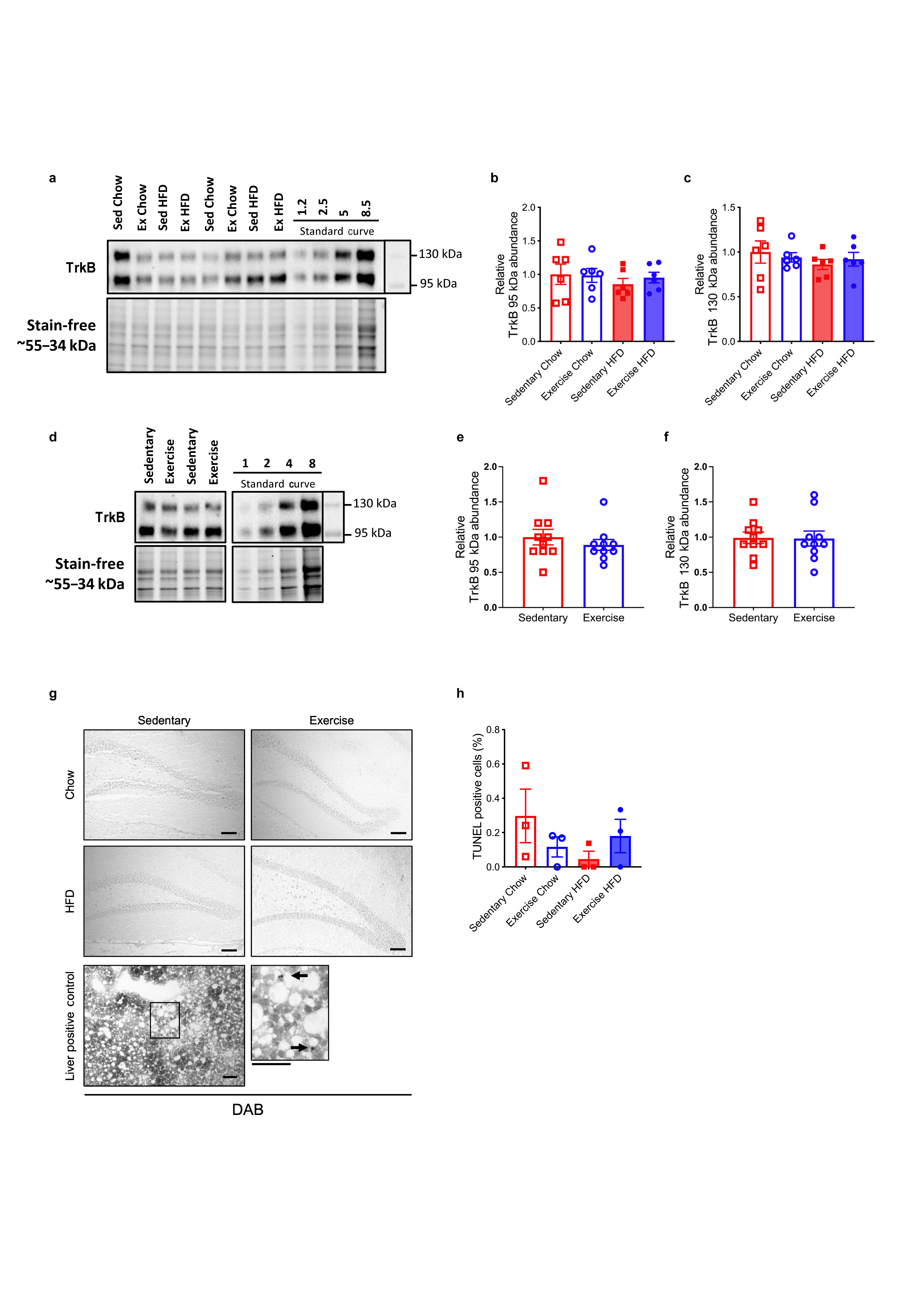


**Supplementary Figure S3** Proteomic analysis undertaken on the hippocampi of the HFD exercise vs sedentary groups. (a) Principle component analysis (PCA) of protein abundance for sedentary (red) and exercise (blue) samples. (b) Volcano plot of differently expressed proteins for HFD exercise vs HFD sedentary. No proteins were significantly different with FDR filtering with a *P*-value of 0.05. (c) Top 10 significantly increased proteins in the exercise vs sedentary groups when using FDR = 1. (c) Top seven significantly enriched pathways. In total, 411/5532 proteins were increased in the exercise group compared with the sedentary group when FDR = 1.


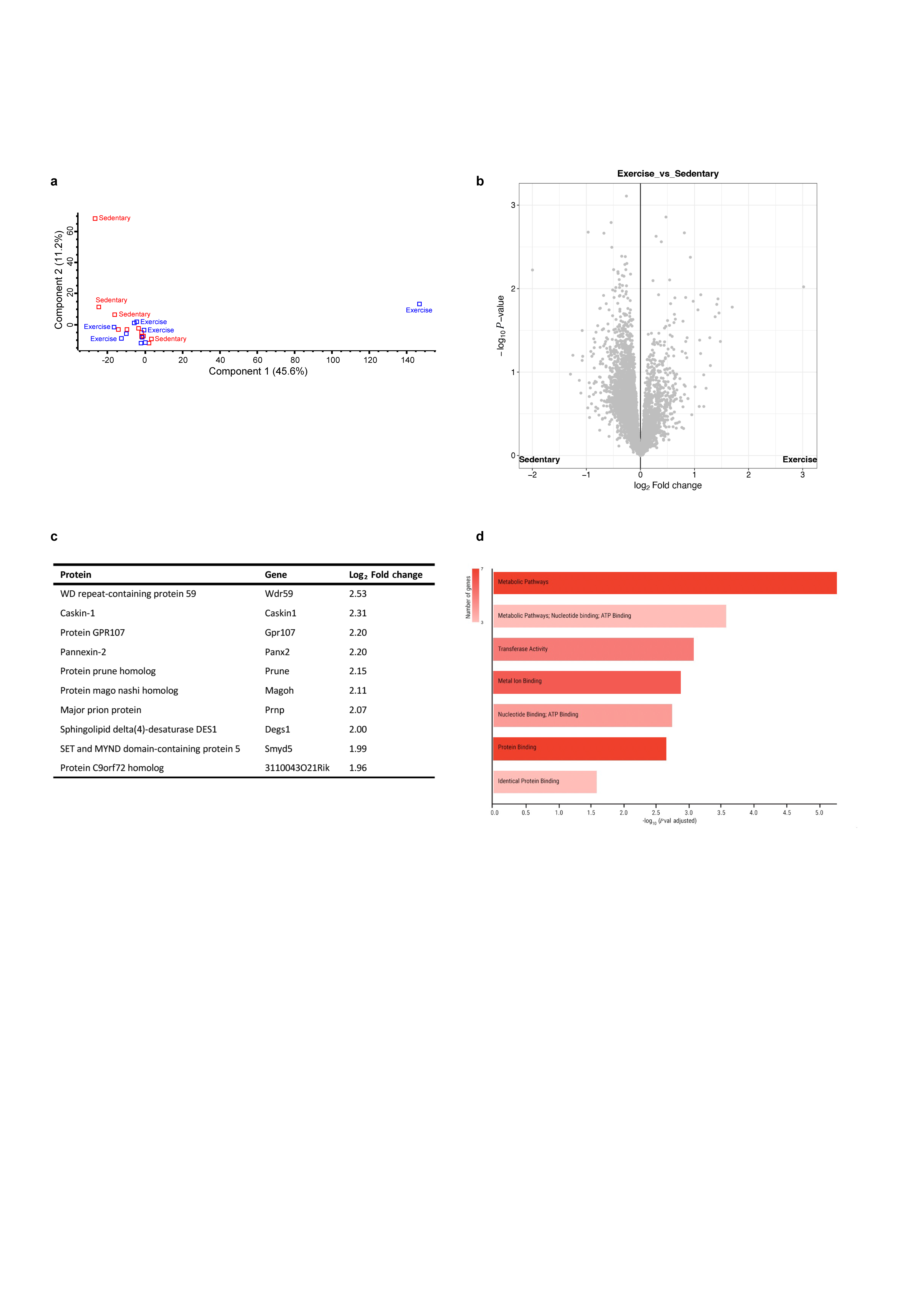

Supplement: load043_suppl_Supplementary_Figures_S1-S3 [file load043_suppl_Supplementary_Figures_S1-S3.docx]
