# Supplementary figures and images for: Exercise training improves long-term memory in obese mice
Source: Life Metab. 2023 Nov 15;3(1):load043. doi: 10.1093/lifemeta/load043 (PMC11749366; doi:10.1093/lifemeta/load043)

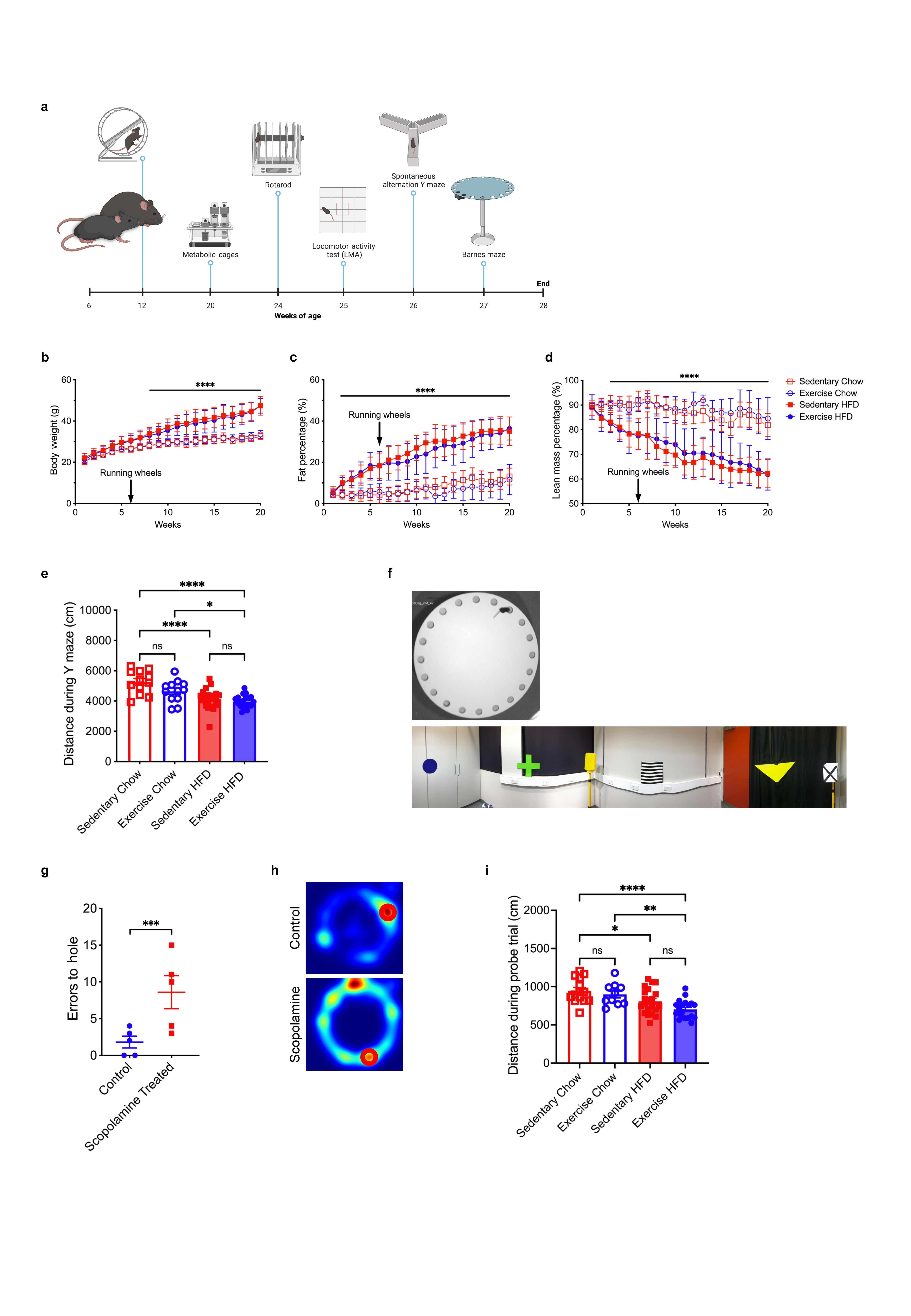

Supplement: load043_suppl_Supplementary_Figure_S1 [file load043_suppl_Supplementary_Figure_S1.tif]

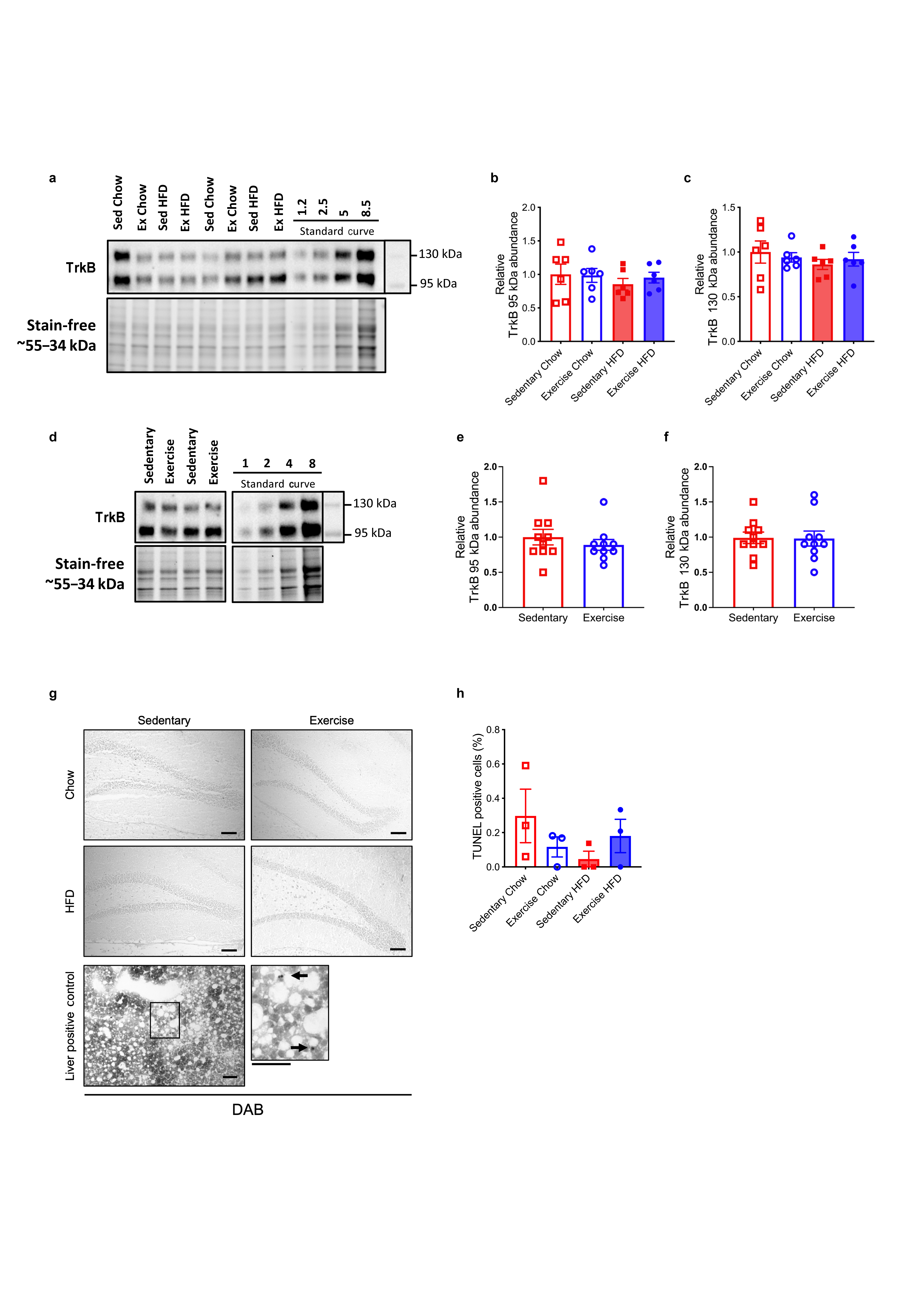

Supplement: load043_suppl_Supplementary_Figure_S2 [file load043_suppl_Supplementary_Figure_S2.tif]

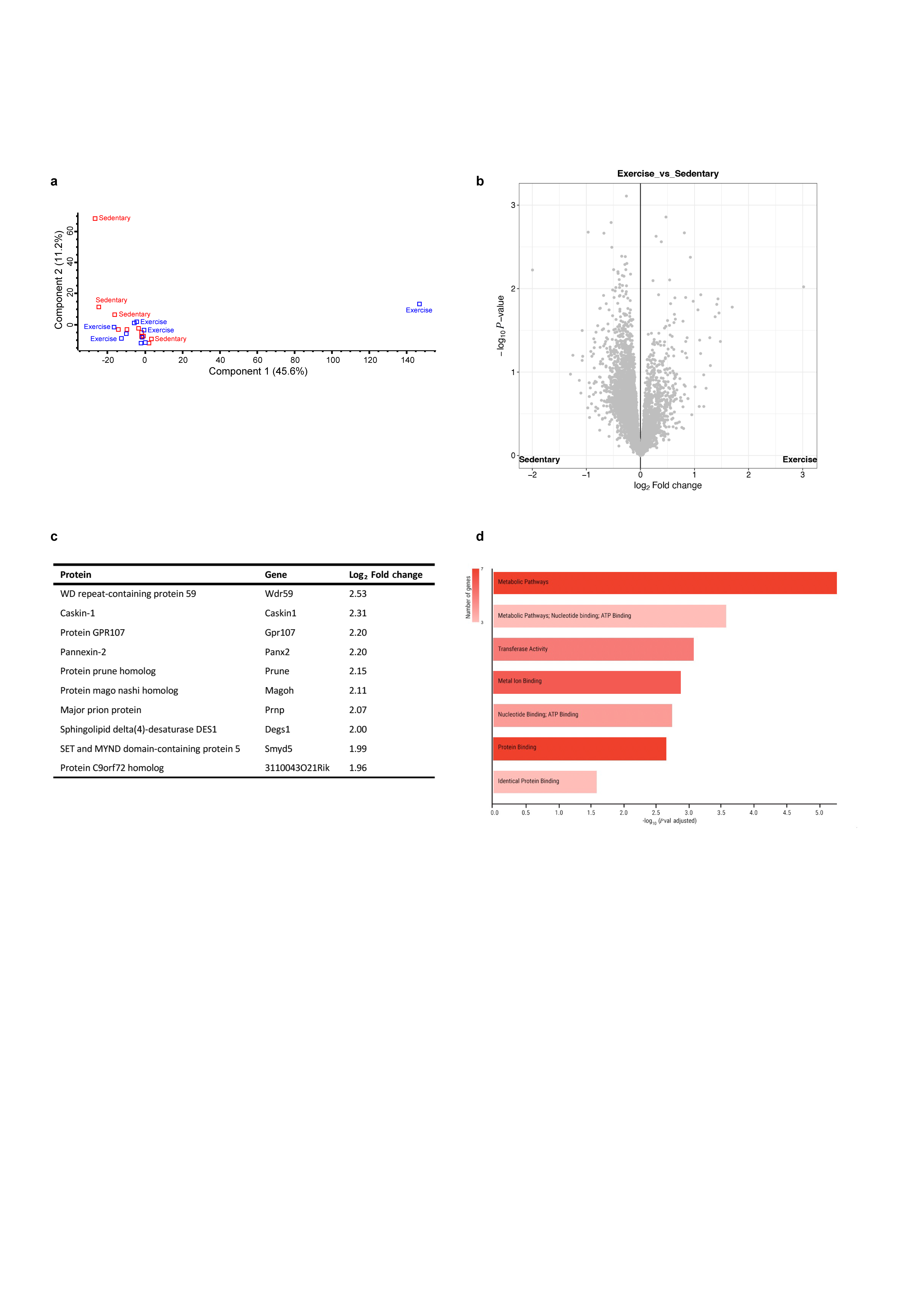

Supplement: load043_suppl_Supplementary_Figure_S3 [file load043_suppl_Supplementary_Figure_S3.tif]
